# Hydralazine-induced antineutrophil cytoplasmic antibody-associated vasculitis with pulmonary–renal syndrome: a case report

**DOI:** 10.1186/s13256-020-02378-w

**Published:** 2020-04-15

**Authors:** Ahmad Al-Abdouh, Abdul Muhaymin Siyal, Hanan Seid, Ammer Bekele, Pablo Garcia

**Affiliations:** 1 Department of Medicine, Saint Agnes Hospital, Baltimore, MD 21229 USA; 2 Section of Critical Care, Department of Medicine, Saint Agnes Hospital, Baltimore, MD USA

**Keywords:** Hydralazine, Drug-induced vasculitis, Pulmonary–renal syndrome, Pulmonary hemorrhage, Glomerulonephritis

## Abstract

**Background:**

Hydralazine is a common vasodilator which has been used for the treatment of hypertension and heart failure. Hydralazine can induce antineutrophil cytoplasmic antibody-associated vasculitis due to its auto-immunogenic capability and one of the very rare presentations is pulmonary–renal syndrome.

**Case presentation:**

We report a case of a 64-year-old African American woman, who presented to our emergency room with shortness of breath, orthopnea, paroxysmal nocturnal dyspnea, leg swelling, fatigue, loss of appetite, cough with clear sputum, and lightheadedness. On admission, she developed acute hypoxic respiratory failure requiring intubation and acute renal failure requiring hemodialysis. A serologic workup was positive for antineutrophil cytoplasmic antibody, antinuclear antibody, anti-histone, anti-cardiolipin IgM, and anti-double-stranded DNA antibodies. A renal biopsy was done due to persistent deterioration in kidney function and demonstrated classic crescentic (pauci-immune) glomerulonephritis. Hydralazine was empirically discontinued early in the admission and she was started on corticosteroids and cyclophosphamide following biopsy results. She was clinically stable but remained dependent on hemodialysis after discharge.

**Conclusion:**

Hydralazine-induced antineutrophil cytoplasmic antibody-associated vasculitis with pulmonary–renal syndrome is a rare occurrence. In the setting of hydralazine use, multiple positive antigens, and multisystem involvement, clinicians should consider this rare condition requiring prompt cessation of offending drug, early evaluation with biopsy, and contemplate empiric immunosuppressive therapy while biopsy confirmation is pending.

## Introduction

Hydralazine is a direct-acting vasodilator which was initially discovered as a treatment of malaria and has been used for the treatment of hypertension and heart failure since the 1950s [1, 2]. Although it has largely been replaced by newer antihypertensive drugs with fewer side effects, hydralazine is still widely used in developing countries due to its lower cost [2]. Hydralazine-induced antineutrophil cytoplasmic antibody (ANCA)-associated vasculitis, while well established in the literature, remains a rare phenomenon; pulmonary–renal syndrome is the most severe presentation of ANCA-associated vasculitis due, predominantly, to renal vasculitis and the need for aggressive management. Fortunately, this severe clinical presentation with pulmonary–renal syndrome is even more infrequent [3–5]. Early diagnosis of hydralazine-induced ANCA-associated vasculitis followed by discontinuation of the drug with prompt medical management will result in better clinical outcomes. To the best of our knowledge, there are 25 reported cases of hydralazine-induced ANCA-associated vasculitis with pulmonary–renal syndrome [6].

Below, we present a case of a 64-year-old woman with history of hypertension on hydralazine who presented with hypoxic respiratory failure and acute renal failure requiring hemodialysis.

## Case presentation

A 64-year-old African American woman with history of hypertension and heart murmur, presented to our emergency room with acute onset of shortness of breath, orthopnea, paroxysmal nocturnal dyspnea, and cough with clear sputum of 4 days’ duration. She also complained of fatigue, loss of appetite, bilateral leg swelling, and lightheadedness of 1-week duration. She denied fever, chest pain, recent hospitalizations, new medications, or recent travel history. Her home medications included hydralazine, amlodipine, losartan, metoprolol succinate, amiodarone, aspirin, gabapentin, cetirizine, trazodone, zolpidem, cyclobenzaprine, chlorzoxazone, fluticasone, docusate sodium, pantoprazole, ferrous sulfate, ibuprofen, and pravastatin.

On presentation, she was afebrile with a blood pressure of 158/76 mmHg, heart rate of 90 beats per minute, and pulse oxygenation of 96% on room air. A physical examination in our emergency room revealed mild respiratory distress, pale conjunctiva, and bilateral lower limb edema. A heart examination demonstrated holosystolic murmur at apex, grade 3/6, and non-radiating. A lung examination demonstrated bilateral diffuse coarse rales, worse on the right side. The rest of the physical examination was unremarkable.

**Fig. 1:**
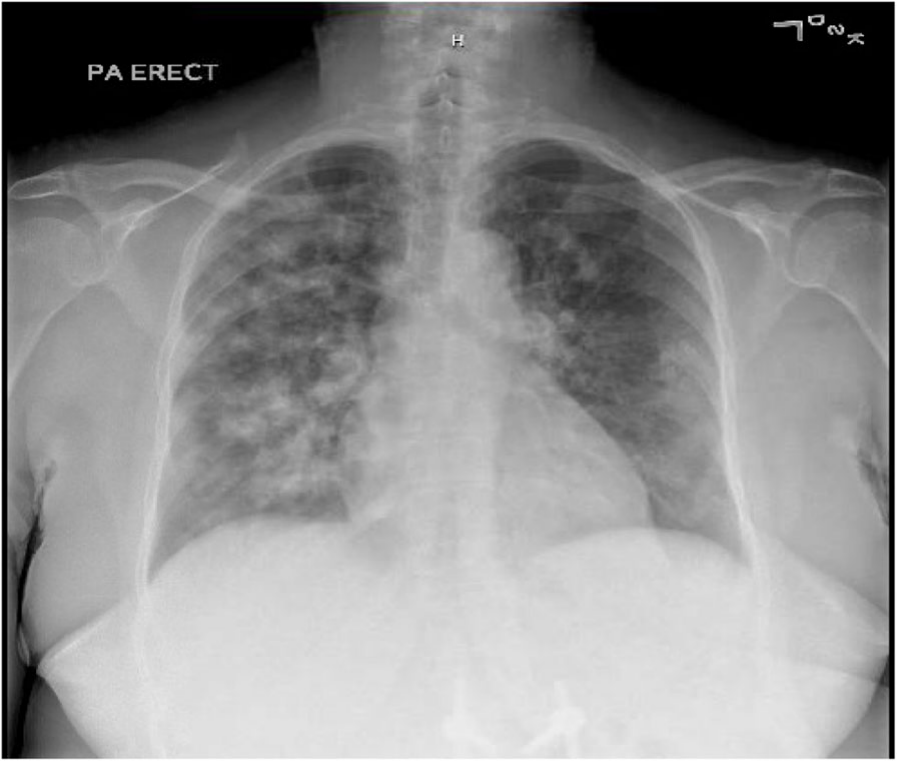
Chest X-ray shows diffuse nodular densities bilaterally but more prominent on the right lung

**Fig. 2:**
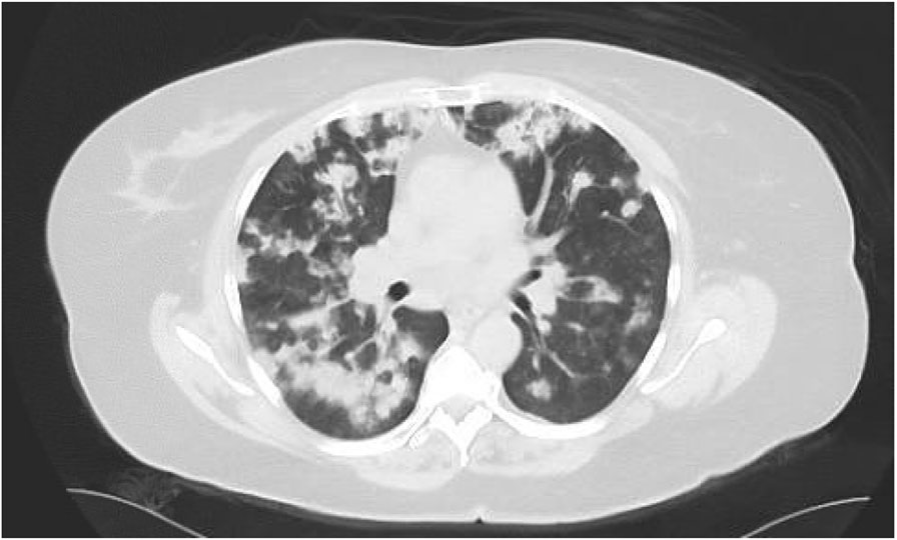
Chest computed tomography shows bilateral irregularly shaped nodules asymmetric on the right with peripheral and pleural-based distribution

Laboratory findings on admission revealed: hemoglobin, 6.9 g/dL (baseline 12 g/dL); white blood cells (WBC), 5.9 × 10^9^/L; platelets, 267 × 10^9^/L; serum creatinine, 4.9 mg/dL (baseline 0.9 mg/dL); erythrocyte sedimentation rate (ESR), 124 mm/hour; C-reactive protein (CRP), 169 mg/L; and urine analysis, 3+ blood and 1+ protein. A chest X-ray **(**Fig. 1**)** revealed diffuse nodular densities bilaterally but more prominent in the right lung. A chest computed tomography (CT) **(**Fig. 2**)** showed bilateral irregularly shaped nodules more on the right with peripheral and pleural-based distribution. A renal ultrasound showed a right renal cyst with mild complexity and left simple renal cyst but there was no hydronephrosis or calculi. An echocardiogram revealed ejection fraction of 65–70% and mild aortic stenosis; there were no valvular vegetations. Table 1 shows the differential diagnoses of this case.

**Table 1:** Differential diagnoses of the case

Differential diagnoses	
Hydralazine-induced ANCA-associated vasculitis with pulmonary–renal syndrome	
Acute heart failure with pulmonary edema and cardiorenal syndrome	
Acute renal failure with pulmonary edema and uremic hemoptysis	
Respiratory tract infection with prerenal renal failure and/or postinfectious GN	
Systemic lupus erythematosus	
Cryoglobulinemic vasculitis	

*ANCA* antineutrophil cytoplasmic antibody, *GN* glomerulonephritis

She was treated with intravenously administered fluids, broad-spectrum antibiotics, and given a unit of packed red blood cells. A serologic workup was requested and hydralazine 100 mg, which she used to take three times daily for the past 8 years, was stopped given systemic nature of the disease and suspicion of hydralazine-induced ANCA-associated vasculitis. On day 2 of admission, she developed acute hypoxic respiratory failure and required intubation. Bronchoscopy was done and revealed diffuse blood lining her bronchi but not occluding it. Her kidney function continued to deteriorate, and she was started on hemodialysis. A kidney biopsy was also performed given the severe acute kidney failure with unclear etiology. Antinuclear antibody (ANA) titers were more than 1:640 (reference range, negative) with diffuse pattern; perinuclear ANCA (p-ANCA) titers were 1:2560 (reference range < 1:20, ARUP Laboratories, Utah, USA); myeloperoxidase (MPO) antibody was positive at 40 AU/mL (reference range 0–19); and serine protease 3 IgG was 383 AU/mL (reference range 0–19). Histone IgG antibody was 6.5 units (reference range 0–0.9); anti-double-stranded DNA (dsDNA) titer was 1:10 (reference range < 1:10); anti-cardiolipin IgM antibody was 35 MPL (reference range 0–12) but the IgG was 8 GPL (reference range 0–14); glomerular basement membrane antibody was negative; complement 3 was 75 mg/dL (reference range 88–201); and complement 4 was 15 mg/dL (reference range 10–40). Serum protein electrophoresis and immunofixation electrophoresis were of normal pattern. She was extubated after 2 days and remained clinically stable but dependent on hemodialysis therapy.

**Fig. 3:**
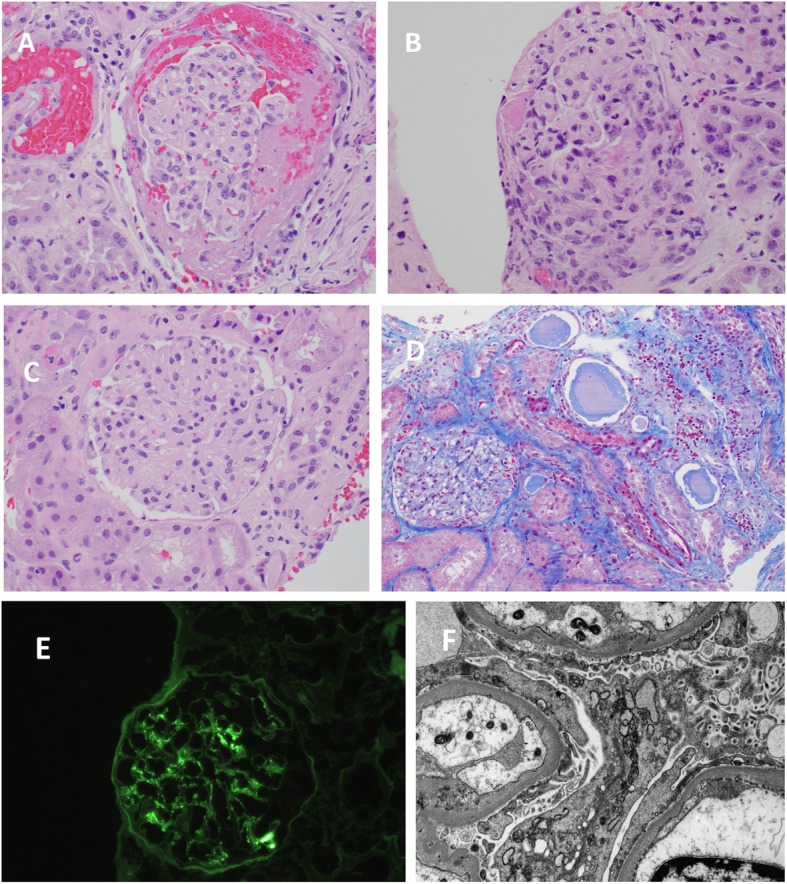
**a** Light microscopy: a glomerulus with segmental fibrin in glomerular tufts, and segmental cellular crescents (four out of 20 glomeruli showed same features). **b** Light microscopy: one glomerulus with global sclerosis. **c** Light microscopy: occasional marginating neutrophils in the remaining glomeruli. **d** Light microscopy: focal mild tubular injury with tubular cell vacuolization, apical blebbing, focal dilation, with some proteinaceous casts and sparse red blood cells casts. The interstitium has mild focal inflammatory infiltrate. There is mild tubular atrophy and interstitial fibrosis. **e** Immunofluorescence: segmental granular glomerular staining for immunoglobulin 2, C3, and trace stain for kappa and lambda. **f** Electron microscopy: a glomerulus with a cellular crescents and some fibrin

A renal biopsy **(**Fig. 3**)** showed focal crescentic glomerulonephritis, with segmental deposits by immunofluorescence, which were not evident by electron microscopy. These changes were suggestive of pauci-immune process related to ANCA-associated vasculitis and ruled out lupus nephritis. She was started on pulse intravenous steroids therapy for 3 days and then on prednisone 60 mg daily in addition to cyclophosphamide 75 mg twice daily.

On follow up after 3 months, she committed adherence to her medications, she was weaned off steroids and cyclophosphamide, and she was weaned off dialysis. Rituximab was started for maintenance therapy. Table 2 shows the timeline of events related to this case.

**Table 2:** Timeline of events

Timeline	Events
Day 0	• Admitted with hypoxia and acute renal failure (on nasal cannula)
Day 1	• Worsening of acute hypoxic respiratory failure (on high-flow nasal cannula)
Day 2	• Worsening of acute hypoxic respiratory failure (intubated)
	• She underwent bronchoscopy
	• She became anuric
	• She was started on pulse dose steroids
Day 3	• She underwent kidney biopsy
	• She was started on intermittent hemodialysis
	• She was started on cyclophosphamide
Day 14	• She was discharged to subacute rehabilitation facility on cyclophosphamide and prednisone (tapering dose)
	• She was scheduled for out-patient intermittent hemodialysis three times per week (polyuric)
Day 45	• Cyclophosphamide was stopped
Day 82	• She was weaned off dialysis (last session)
Day 113	• She was given the first dose of rituximab for maintenance therapy
Day 297	• She finished her tapering steroids course
	• She was given the second dose of rituximab for maintenance therapy
	• Kidney function is stable (GFR 49 mL/minute/1.73 m^2^)

*GFR* glomerular filtration rate

## Discussion

Hydralazine-induced vasculitis has an incidence of 5.4% in patients using 100 mg/day and 10.4% in patients using 200 mg/day for more than 3 years’ duration. The incidence is higher in patients who are slow acetylators [7]. ANCA-associated vasculitis are often idiopathic, however, infections and drugs like hydralazine are the most common triggers for the onset of disease process. It is still unclear how hydralazine can induce vasculitis, and this might be multifactorial. One hypothesis is that hydralazine decreases DNA methyltransferase expression and induces autoimmunity by inhibiting extracellular signal-regulated kinase (ERK) pathway signaling and that may be responsible for disrupting the suppression of proteinase 3 (PR3) and MPO [8]. A second hypothesis is that hydralazine is metabolized by MPO released from activated neutrophils to form reactive intermediates that result in the forming of anti-MPO antibody [9]. It was also reported that hydralazine-induced vasculitis is more common in slow acetylators since hydralazine acetylation will be slower and that gives more chance for a break in tolerance [10]. The risk for developing hydralazine-induced ANCA-associated vasculitis increases with longer duration and higher doses of hydralazine therapy especially in slow acetylators, female patients, and patients with history of thyroid disease [11, 12].

The combination of anti-histone antibody, very high titer of MPO and/or PR3 ANCA, and pauci-immune glomerulonephritis, support the diagnosis of hydralazine-induced ANCA-associated vasculitis in the appropriate clinical setting [13]. The Naranjo nomogram [14] was used to assess the likelihood of association of adverse drug reaction to hydralazine, and the score was 7, which indicates probable association. A rechallenge test to further solidify a causal relationship could not be performed due to the severity of the reaction. A diagnosis of hydralazine-induced ANCA-associated vasculitis in this case was established based on the following: positive anti-histone, very high MPO-ANCA titers, positive anti-cardiolipin IgM, absence of skin and musculoskeletal involvement, the results of the kidney biopsy with pauci-immune pattern typical of ANCA-associated vasculitis, and clinical improvement upon discontinuation of hydralazine. What distinguishes this case from other reported cases is the presence of low titer of anti-dsDNA, which is usually associated with systemic lupus erythematosus (SLE) vasculitis, even though the multi-antigenicity and renal biopsy support the diagnosis of hydralazine-induced ANCA-associated vasculitis.

Renal outcomes for this condition in published cases have been variable, with one case series reporting full recovery in all patients [11] and others reporting around one third to half of patients requiring long-term hemodialysis despite immunosuppressive therapy [15, 16].

There are no large-scale studies to establish the most effective treatment, but expert consensus includes discontinuation of hydralazine with immunosuppressive therapies based on disease severity. Corticosteroids with cyclophosphamide or rituximab and therapeutic plasma exchange may be considered on an individual basis especially in cases associated with pulmonary–renal syndrome. It is important to warn the patient about the future use of the offending drug and include it in their adverse reaction list [6].

## Conclusion

Hydralazine-induced ANCA-associated vasculitis with pulmonary–renal syndrome differs from idiopathic ANCA-associated vasculitis in its severity. Our patient had multiple risk factors (female and prolonged high-dose exposure) for developing this drug-induced condition. The diagnosis was based on serology, absence of skin manifestation, bronchoscopy, renal biopsy results, and improvement upon discontinuation of hydralazine. High clinical suspicion, early diagnosis with drug discontinuation, and aggressive management are necessary to achieve a favorable patient outcome.

## Data Availability

All data and material are available upon request.
